# The challenging reality of the clinical learning environment at Damascus University Faculty of Dental Medicine in Syria: a qualitative study

**DOI:** 10.12688/mep.19564.2

**Published:** 2023-07-31

**Authors:** Ghaith Alfakhry, Khattab Mustafa, Bashar Jazayerli, Khaled Alhomsi, Issam Jamous

**Affiliations:** 1 Education Quality and Scientific Research Office, Al-Sham Private University, Damascus, Damascus Governorate, N/A, Syria; 2 Faculty of Dental Medicine, Damascus University, Damascus, Damascus Governorate, N/A, Syria; 3 Program of Medical Education, Syrian Virtual University, Damascus, Damascus Governorate, N/A, Syria; 4 Department of Restorative Dentistry and Endodontics, Faculty of Dental Medicine, Damascus University, Damascus, Damascus Governorate, N/A, Syria; 5 Department of Biomedical Sciences, Al-Sham Private University, Damascus, Damascus Governorate, N/A, Syria; 6 Department of Prosthodontics, Faculty of Dental Medicine, Damascus University, Damascus, Damascus Governorate, N/A, Syria

**Keywords:** Clinical professional training, postgraduate dental education, clinical learning environment, Damascus University, Syria, semi-structured interviews, qualitative study

## Abstract

**Introduction:** In Syria, specialist dentists undergo five years of undergraduate education and four years of postgraduate education. In the latter, students engage in treating complex cases as part of their professional training. This study aimed to obtain in-depth qualitative understanding of the clinical learning environment at Damascus University Faculty of Dental Medicine, Syria.

**Methods:** Semi-structured interviews were held with eight postgraduate dental students at Damascus University Faculty of Dental Medicine. The faculty has eight clinical departments; therefore, a single participant was purposively sampled from each department. The male-female ratio of the eight interviewed participants was 1:1. All interviews were conducted between 26 ^th^ April 2020 and 8 ^th^ January 2021. Data was analyzed inductively using reflective thematic analysis. Pragmatic saturation was discussed during the analysis and the authors made an interpretative judgement to stop data collection at the eighth interview.

**Results:** Major themes which emerged covered different aspects of the clinical learning environment such as clinical training, social interaction and assessment procedures. Faculty’s negligence of their teaching duties was one of the most recurrent themes. In clinical training and due to faculty inaccessibility, students had to rely on themselves or their senior peers in training. The social climate was perceived negatively and assessment was described as unfair and biased.

**Discussion:** The findings of this study showcased the continuing deterioration of the clinical learning environment at Damascus University. It is hoped that these findings will encourage decision makers to introduce a comprehensive reform that addresses the curriculum, teaching practices and assessment procedures in clinical professional training.

## Introduction

To health profession educators, the clinical learning environment (CLE) is an area of utmost importance that plays a pivotal role in education outcomes ( Irby, 2018; Nordquist *et al*., 2019). In the past couple decades, the learning environment (LE) has been one of the main areas of focus of healthcare education research and development. The myriad of research in this area and lack of consensus on a single definition hints at the complexity of the LE as a concept ( Nordquist *et al*., 2019; Schönrock-Adema *et al*., 2012). Dental education literature illustrates that the LE encompasses: ‘clinical work’, ‘assessment procedures’, ‘social interactions’ and ‘ethical climate’ ( Divaris *et al*., 2008). The CLE is especially emphasized in postgraduate professional education, where learning overlaps with clinical work ( Kilty *et al*., 2017).

At the Josiah Macy Jr. Foundation consensus conference, the following definition of the learning environment has been proposed: “Learning environment refers to the social interactions, organizational culture and structures, and physical and virtual spaces that surround and shape the learner’s experiences, perceptions, and learning ( Gruppen *et al*., 2018).” Gruppen *et al*. has proposed a conceptual framework in which key elements of the LE has been highlighted ( Gruppen *et al*., 2019); the LE has been framed into five interwoven components that form two conceptual dimensions: the psychosocial dimension and the material dimensions; the psychosocial dimensions having three components: personal, social and organizational and the material dimension is comprised of the physical and virtual spaces. In the psychosocial component, the personal component describes how learners engage with personal growth, interact with LE, and form their own perceptions of the LE; the social component describes the relationships and social dynamics occurring within the LE such as the peer-to-peer, learner-to-faculty, and learner-to-patient relationships. The organizational component focuses on how individuals interact with organizational culture, practices, policies such as teacher control and the curriculum. The second dimension of the LE, the material dimension, encompasses the physical spaces such as buildings, hospitals, clinics and other sites that student use for learning and practice. Learning, nowadays, does not only occur within physical spaces but also in virtual ones such as electronic LEs and digital learning aids ( Gruppen *et al*., 2019).

In dental education, a positive LE is characterized by fairness and respect ( Divaris *et al*., 2008); transparency between students and faculty is emphasized in terms of the desired learning objectives and assessment procedures. An ideal LE should foster collaborative culture values within students. Further, students’ personal development is as important as their professional development. Assessment wise, faculty should adopt for-learning assessment mentality rather than the traditional paradigm of ‘assessment drives learning’. Feedback should be conversational not didactic, constructive rather than demoralizing, and students should feel free to express their views.

Damascus University (DU) Faculty of Dental Medicine is the largest and oldest governmentally funded educational dental institution in Syria. Its curricula have been previously described as conventional and teacher-centered ( Kayyal & Dashash, 2010). In 2007, Damascus University and a team of external experts attempted to introduce a reform to modernize the current system and change the rules that govern the delivery of teaching and learning. This reform targeted healthcare-related schools including Faculty of Dental Medicine and quantitively examined undergraduate students and faculty members expectations of the education system. However, by the end of 2009, all attempts to develop and reform education at the Faculty of Dental Medicine were deemed unsuccessful and no progress was made. As per the leaders of this reform, the main obstacles which faced this transformative plan were: centralized decision making, lack of motivation for change, problematic University hierarchy, inappropriate and obstructive rules and regulations, lack of awareness of the competencies needed of faculty members to achieve curriculum development and reform ( Kayyal & Gibbs, 2012).

After 12 years of war, the education quality at the Damascus University Faculty of Dental Medicine continues to deteriorate in the light of political and economic turmoil ( Shehada *et al*., 2022; Shehada *et al*., 2023). At the postgraduate level, there is no previous record of the education outcomes, teaching strategies, nor assessment processes conducted at DU Faculty of Dental Medicine. It’s important to evaluate these core elements of the CLE in what can be described as a strictly conventional educational system which lagged behind and failed to reform its policies in accordance to modern health profession education standards. The findings of this study are important for the institution itself as well as other dental schools that share core characteristics with Damascus University Faculty of Dental Medicine (war-affected, under-resourced, conventional teaching practices, teacher-centered curriculum). Hence, it is this study’s goal to provide the first evaluation of the reality of the clinical learning environment at DU Faculty of Dental Medicine as perceived by postgraduate students.

## Methods

### Ethics

Ethical approval was granted by the ethical committee at Damascus University Faculty of Dental Medicine on 17 ^th^ February 2020 (approval number: 76559). Written informed consent was obtained from all participants, and all methods were carried out in accordance with relevant guidelines and regulations. This study is reported in accordance to the Standards for Reporting Qualitative Research guidelines ( O’Brien *et al*., 2014).

### Study design

This study adopted an interpretivist paradigm in which reality is viewed as subjective, multiple and socially constructed. Therefore, semi-structured interviews were undertaken with postgraduate students in all clinical departments, interviews were transcribed and analyzed inductively using reflective thematic analysis. This study followed a descriptive phenomenological approach to address the research question which is phrased as follows: how do postgraduate dental students perceive the clinical learning environment?

### Settings and participants

DU Faculty of Dental Medicine offers nine academic masters of science courses. Those students are accepted into the postgraduate program based on their grade point averages (GPAs) and their grade at the national dental exam. There are about 8 to 15 slots at each master’s program, making them very competitive in comparison to over one thousand graduates who apply ( Alfakhry *et al*., 2023a). Dental education in Syria requires five years undergraduate education and four years of postgraduate education. Undergraduate dental education is divided into three preclinical years and two clinical years ( Damascus University, 2007). At the postgraduate level, the first two years are clinical and module-based, and students are required to treat patients in these two years as part of their clinical professional training; the 3 ^rd^ and 4 ^th^ years are dedicated for conducting a research project. All departments have a clinical-work aspect with the exception of the Department of Oral Pathology which was excluded. These 8 clinical departments cover the following specialties: Orthodontics, Maxillofacial Surgery, Oral Medicine, Pedodontics, Endodontics and Restorative Dentistry (as one department), Removable Prosthodontics, Fixed Prosthodontics and Periodontology.

All interviews were conducted between 26 ^th^ April 2020 and 8 ^th^ January 2021. At this time, all participants were early year-three postgraduate students, with the exception of one participant who was in early year-four. In total, eight postgraduate participants were purposively sampled for the study from each of the eight above-mentioned departments; all of whom consented to participate. Purposive sampling was used and contact details were obtained by KM as he was an acquaintance of all study participants. As for the participants sex (biological distinction as self-reported), four participants were females and four were males and the sample age range was between 25 and 26 at the time of conducting the interviews. For the determination of sample size, pragmatic saturation was discussed upon finishing analyzing eight interviews by researchers and an interpretative judgement was made that the richness of the collected data are enough for the goals of the current research ( Braun & Clarke, 2021). All participants were Syrians who lived their entire life in Syria and graduated from DU Faculty of Dental Medicine. The interviews were conducted in Arabic in a one-to-one, face-to-face manner. Interviews were held in the place that the interviewee and the interviewer found convenient, most of these places were either coffee houses or at the faculty in a private room. When the interview took place in coffee houses, it was ensured that the conversation was private and nobody was listening, so that the interviewee felt comfortable sharing sensitive information.

### Personal characteristics of the interviewers

The research team who conducted the one-to-one interviews were GA and KM, both of which are male and graduated as dentists from Damascus University in 2020, 2018 respectively and both have a Master’s degree in Medical Education. This facilitated access to, and rapport establishment with, the interviewees.

### Relationship with participants

All participants were previous colleagues of the research team member KM, who in turn introduced them to GA. A friendly colleague-relationship was established prior to conducting the interviews. The participants were initially informed that research goal was to explore the general condition at their respective departments. At the end of each interview, the specific research goal-evaluating the clinical learning environment was communicated with the interviewees, and any final comments were elicited. In terms of interviewers’ characteristics, both are interested in improving the quality of dental education at Damascus University and are motivated to reveal the academic, clinical and social hurdles dental students face during their postgraduate education.

### Data collection

Prior to commencing the interviews, an interview protocol ( Alfakhry & Mustafa, 2023) was designed and piloted on a single participant. The average duration for the interviews was about 45 minutes. All interviews were audio recorded and transcribed verbatim in Arabic by BA and checked for accuracy by GA. The interview schedule included four main parts:

1. Clinical training
2. Clinical assessment and evaluation
3. Students’ perception of the role of teaching staff
4. Students’ perception of their peers

Participants were engaged in in-depth discussions regarding the CLE. They were also encouraged to reflect and give specific critical incidents which illustrate their learning experience. Detailed description about these critical incidents was elicited from participants. This description included the observed behavior of those involved, participants’ speculation of the reason behind this certain behavior and their attitude towards it. Repeat interviews were not conducted and interview transcripts were not returned to participants for comments. However, the final study report was shared with participants.

### Data analysis

GA was responsible for data coding. To counteract the fact that the main interviewers and analysts GA could make some analytical assumptions due to having similar educational background to participants, codes and themes were double-checked by KM and IJ who have diverse educational experience in medical, dental and health-sciences education. All transcripts were in Arabic during the coding and analysis process; transcripts were not translated into English to ensure accurate understanding of transcripts by the analyst (GA), and avoid semantic loss due to translation from a language into another as the context is culture bound ( James, 2002). Interviews were deidentified during transcription process. An iterative process of collection and analysis took place. Upon the identification of potential themes, more probing questions were added to explore the themes in more depth. For example, more probing questions were added regarding the role of supervisors in teaching and their availability and accessibility during training time. Codes were identified inductively, as in induced from the interviews and transcripts; line by line coding strategy was implemented.

The identified themes were derived from the data in an inductive manner where data determined the themes. MAXQDA 2020 ( VERBISoftware, 2020) was used to conduct thematic analysis with simple counting methods. An open access app with similar function to MAXQDA is QDA Miner 6 ( ProvalisResearch, 2020). All presented quotations underwent a double translation process from Arabic to English and vice versa. Some identifying information was redacted such as those the research team thought to be revealing to certain departments, faculty members, and/or students. Some information was redacted upon direct request from the participants. All authors checked the consistency between the data presented and their interpretation. The credibility of the findings was checked by triangulating the reports of different participants. Certain anomalies and diverse cases were also reported and discussed. Even though there was an adequate representation of both males and females, sex-related differences among participants were negligible during the analysis and therefore were not included in the results. For presentation purposes, each participant was assigned a random number from 1 to 8; in terms of sex distribution, participants no. 1,3,5,7 are males and the rest are females.

## Results

Eight major themes emerged from participants' educational experience at their departments.

### Faculty’s negligence of their teaching duties

The most prominent theme was faculty’s negligence of their teaching duties. Participants find this very problematic and obstructing to their clinical learning activities. Negligence took various forms as reported by participants. One of the most common ones are the physical unavailability of faculty members, being busy with non-teaching related activities or uninterested in teaching students.

**Participant 5**: *We have 8 professors. One of them should be present at the department each day. No professor is showing up. Even the one who is responsible of supervising clinical work that I told you about is also unavailable. If available, they stay until a specific hour with no real supervision on the procedure that we, students, are doing. They stay at their desks; we bring our logbook for them to sign and we work without any intervention from them... To be blunt with you, their presence at the department is just for the academic title more than being professors responsible for the educational process. They just want to write at their private clinics banner “a teaching staff at the Faculty of Dental Medicine, Damascus University”, but in actuality, they have no presence.*

The phrase “ *without any intervention*” expresses frustration of the participant with the disinterest and negligence faculty display in clinical rounds. “ *just for the academic title*”, “ *write at their ….teaching staff*” highlight the perceived egotistical motives of faculty for being teachers. Participants repeatedly emphasized the importance of having active professors around and they were deeply troubled for not having this essential component in their clinical training.

**Participant 5** *: I realize how important it is for professors to engage in teaching us, to give us a little bit of their time, just a little bit… I could gain a lot of experience from our professors if they were there, I don’t know why they do what they do?! Do they have more important things in life than this?! I think if they have more important things, they should stop teaching, because teaching, takes time, it takes devotion.*

The phrase “ *just a little bit*” shows desperation. “ *I don’t know why”,“they should stop teaching*” implies participant’s inability to find an excuse for faculty’s negligence of their responsibilities. Another participant explained that when professors are available, they only check on students if requested to. This was echoed by 7 out of 8 participants.

**Participant 2**: *Professor x checks on us a bit, but not much, you need to chase them if you have a problem. I don’t think there is a professor that checks on you without being asked to.*

Another identified form of learner neglect was teacher’s taking over in the treatment phase. A participant explained that when he calls for the professors to help guide him in managing a complication in his clinical case, the professor just deals with the complication himself quickly ignoring the learning experience the student is seeking.

**Participant 1**: ..an *incident with a professor regarding broken instruments (in the tooth canal). While you are working (on the case), he comes in a rush. You want to observe (what he is doing to deal with the complication). The microscope is supposed to have to two pairs of binoculars, one for the professor and one for the student so that the student can learn. We don’t have that despite its importance. What happens is that the professor holds the tool to free the instrument in the canal, but you can’t see, what’s the use?!. So he comes quickly, frees the instrument and sometimes I’m not doing anything except for standing idle.*

The statements “ *what’s the use?*”. “ *I’m not doing anything except for standing idle*” indicates students’ disappointment in not being able to learn from his case due to the teacher’s main focus on treating rather than teaching. In this case the teacher took over because he was busy, however, that’s not always the case. In another department, a participant explained that faculty sometimes *steal rare cases* to hone their own clinical skills.

**Participant 3:** *Prof x likes to steal nice cases*

**Interviewer:** *for whom?*

**Participant 3:** *for himself*

**Interviewer:** *at the faculty or outside?*

**Participant 3:** *No, at the faculty…for example, in bone-splitting or sinus lift operations he likes to take over, you know. Prof y, doesn’t intervene although he is qualified, whereas, Prof x likes to take over. Prof a, likes to take over when a student is taking too long…Professor z is not very skillful in procedures so he started learning on our account. Students bring patients and he would take over and mess it up…if I become a professor like him, I might steal cases from students and try my hands on them, you can’t blame them completely for it, but why the mess ups (cause a complication)?! They shouldn’t be involved in a case they can’t handle.*

The previous participant response gives various examples of professors’ interventional/non-interventional behavior in the operating theatre. The participant comments such as “ *I might steal cases*” and “ *can’t blame them*” indicate student’s attempt to find an excuse for professors’ theft of their cases but “ *why the mess ups?!*” show that student was unhappy when professors can’t handle a case they decided to take over in.

### Relying on oneself to attain clinical skills

Another major theme that emerged from the interviews is reliance on oneself in clinical training. Participants reported that it’s up to the students to decide what to do and what not to do. Students can treat as many cases if they wish, no matter how complex the case could be. They could attend and go to the clinic, and they can choose not to, they can treat 40 cases during the semester or treat 10. Some students reported positive attitude towards being independent and not being monitored by professors. The following response was echoed by 5 out of 8 participants:

**Participant 2:** *What I like about this department is that nobody watches you, you can bring more than one case to try your hands on. There are no limitations to treat other kind of cases which are out of the scope of your specialty. You can do an implant although that you are not a surgeon, you can do endo, you can do whatever you want. You can learn, if you really want to learn.*

The phrase “ *nobody watches you*” implies participants’ positive attitude towards the state of not being monitored. In contrast, in another department where there were stricter rules imposed on students’ practice, one participant complained regarding those restrictions.

**Participant 3:** *The negative thing about professor y is that he likes conservative treatments more. For example, in a simple gingival recession, he advises to monitor the case, in a gummy smile case, he advises to inject Botox instead of lip repositioning surgery. We are an educational institute at the end. You want to treat more cases, make more mistakes. So he should approve more cases. I used to like this guy but after he disapproved me numerous cases in the first two years, I started taking my patients to another professor who would approve cases more.*

*“We are an educational institute at the end”, “make more mistakes”* indicate lack of participant's awareness of the institute’s role as a healthcare provider.

There are multiple negative effects of letting students rely completely on themselves in training. Interviewees reported some malpractice that resulted from learning through trial and error and not adhering to evidence-based protocols which, unfortunately, were not provided by the department.

**Participant 2**: *I feel bad for patients; they are being treated as lab rats, and I thank God I’m not in the patient’s shoes.*

“ *Being treated as lab rats*” indicate the experimental learning that takes place on the account of patients’ safety. “ *I feel bad for patients*” and “ *I thank God…shoes*” imply the perceived negative position of the patient.

One participant elaborated that students’ follow treatment protocols that they created based on their own experience, or heard from their peers. This participant emphasized that “ *self-teaching is dangerous*” in clinical settings and reported an incident in which a patient’s life was on the line.

**Participant 8:** *We didn’t start taking lectures on sedation ‘till the second year and they explained to us the criteria and other things, but before that, the volume of malpractice was significant. We had a deeply sedated patient whose life was on the line, his oxygen levels dropped to 60, which is catastrophic, we barely managed to save the patient.*

This response also highlights the risks of experimental learning. Participant 8 went on to explain that they had to take the patient to the hospital and managed to get the case under control. After this incident, participant 8 explained that a certain professor put some rules and protocols to control students’ clinical practice. This case also illustrates the problems students face because of the inaccessibility of teachers.

**Senior peers are the “ *main educational resource*”**

Interviews revealed that because of teacher’s negligence of their teaching duties, students started looking for other sources of guidance such as consulting a senior peer who had more experience. Informal peer-assisted learning was a major theme that characterized postgraduate clinical education. All participants expressed their reliance on senior peer guidance at some level in clinical practice. One participant described them as their “ *main educational resource*”. The following responses were echoed by all participants:

**Participant 1:** *Our seniors who are older by a year are our primary academic resource, our main educational resource.*

**Participant 2:** *Some senior peers teach more than professors…I have a colleague who is 2 years older. This colleague is my go-to consultant in complex cases.*

**Participant 7:** *The last resort when a case becomes too difficult is consulting professors, I try as much as I can to handle it myself…At first, I consult my senior peers, then PhD students, then professors if they were available.*

These responses highlight the pivotal role of senior peers in the teaching process. “ *Some senior peers teach more than professors*” reflects the higher value placed on peer teaching in comparison to that of a faculty.

### Self-centered, competitive social environment

Regarding the social environment, self-centeredness and competitiveness was another theme echoed by most participants. Same-year colleagues had a negative attitude towards each other as they were perceived as self-centered. The following responses were echoed by 6 participants:

**Participant 4:** *There is competition. There are grudges. Don’t be surprised. I see this a lot in our department. I cannot say that about everyone, but the majority don’t like seeing others succeed. They like competition and seizing things for themselves…I don’t trust my colleagues at all…and there is always a hidden motive when someone compliments you.*

The term “ *grudges*” implies that there is persistence of the implicit hostile interactions among peers. “ *there is always a hidden motive…compliments you*” shows the participant’s cynical attitude towards a behavior ( *complimenting*) that is positive on the surface.

### Poorly equipped facilities negatively affect students’ clinical education

The technical capacity of the faculty was another issue reported by students. The status of poorly equipped facilities was a common theme participants described as an obstacle. As per participants report, most departments stopped providing essential dental materials for students. Participant 1 statement in theme 1 of “ *lack of dual binoculars on an endodontic microscope*” is an example when the technical capacity hindered students’ learning. Some appliances such as dental sensors, dental units, and even the operating room were out of service. Participant 3 reported that they were asked to buy plastic bags for the dustbins; it was also reported that they had to clean the clinical department; these activities take from trainee’s valuable training time. Moreover, the operating room at the oral-maxillofacial hospital at the faculty is out of service and this hindered surgery students from observing and doing major surgeries.

**Participant 5:** *The hospital at the faculty is mostly out of service…A major surgery needs an operating room which is mostly out of service. Every couple of months, one operation is conducted. That’s one of the learning obstacles.*

At the Department of Pedodontics, the participant explained that the Special Needs Children Clinic was also out of service, and that the oxygen and nitrous cylinders were not available. This made them unable to practice certain procedures that require deep sedation in order to conduct dental treatment; a very crucial part of their practice as pedodontists.

### ‘Work-focused’ rather than ‘teaching-focused’ clinics

Participants description of the clinic work did not fit into what is called clinical training. It was more ‘Work-focused rather than teaching-focused clinic’ This theme characterized 7 out of 8 departments. Two departments were especially busy, the Department of Restorative Dentistry and the Department of Pedodontics. It was stressed by the interviewees that this busy environment hindered their educational progress. One interviewee explained that they were being treated as workers by the dean office and their professors.

**Participant 1** *:* *One of main things that I dislike about this department is that they force us to treat certain patients. We are forced to treat certain patients who are referred to us by certain people. This takes time and money and you feel you wasted your entire day sometimes…*

The term “ *force*” implies abuse and inappropriate use of power. There are some minor themes and anomalies which are worth mentioning. At each department, there was a teacher or two who seemed to support students in their clinical training. Another minor theme is that the Department of Periodontology was much more structured than other departments. Each day, there was a professor who was “ *forced*” to supervise students during their clinical practice; although it was mentioned that the attending professor leaves early sometimes. Moreover, senior year students and PhD students were responsible for supervising their younger peers at this department.

### Unfairness of the assessment procedures

Participants reported two main methods the faculty used in high-stake exams to assess students’ clinical skills acquisition at the end-of-the-term. As per participants, four departments used a case presentation, three departments used oral interviews and one used both. Both methods reportedly had major issues. First, the case presentation depended largely on the number of cases presented which is not standardized between students nor faculty members, which was problematic for and unfair to students. Students’ grades were based on the number of cases they hand in; some students manage to finish a lot more cases than others, and teachers’ “ *care about quantity more than quality*” as per participant 1. This puts those students who didn’t manage to finish as much as others at a disadvantage. 7/8 participants reported that they tried to standardize the number of cases amongst themselves; however, issues arose in this attempt.

**Participant 7:** *There is a student who told the faculty members that he had finished over 30 cases-he has a private clinic and he has no problem working until 5 pm, the professor after this sent a message that if any student hands in below 20 cases, grades will be deducted. After that, my colleagues said we need to standardize the number of cases, and I refused. Everyone started screaming at me saying that “you want to do whatever you want”. I told them that I worked hard to treat all these patients…*

Concerning using interviews as a high-stake assessment, two participants mentioned that it is *“random*” and “ *luck*” plays a great role in it as students could be either asked very simple or very complex questions.

**Participant 5:** *The interview lasts 5 minutes for each student, OK. During these 5 minutes, the student is graded out of 100. OK. How is the exam process?...It’s comprised of questions written on pieces of paper that you withdraw randomly. Even the questions, they could cover topics such as frenectomy which is one of the basic procedures here at the department or more complicated topics such as radiotherapy and chemotherapy… Even during the assessment process, you open your paper and try to answer your question while professors are chatting amongst themselves, you know?. So you feel like you are talking to a wall. One professor or two could hear you and make fun of you. That’s the evaluation they do. It doesn’t make sense at all.*

“ *Professors are chatting amongst themselves*”, “ *One professor or two could hear you*” hints at the perceived disinterest of professors; “ *feel like you are talking to a wall*” indicates the isolation and exclusion the student experiences when interacting with professors; “ *It doesn’t make sense at all*” implies participants’ confusion over the assessment process.

### Student-professor relationship as an assessment criterion

Participants across all departments identified common factors that affect their grade, these are: their personal relationship with the professor, favoritism, obedience, attendance, number of cases students do and to a lesser degree, the quality of work.

Having powerful connections was also mentioned by participants as an aiding factor in students’ success. A powerful connection was defined by participants as someone in a high position, or having a parent or a relative who are close to the faculty. The following response was echoed by all participants:

**Participant 8:** *By using your connections, you can obtain grades and become one of the top students*

Participant 8 also reported a case of a student who did not meet the course requirements -the minimum number of cases-but still passed due to having a certain influential connection that made this possible. Participant 8 contrasted this case with another colleague who was described as committed but had to leave for a month due to urgent matters; although a professor permitted her to leave, the student failed because she finished 20 cases in comparison to her peers who managed to finish around 40 cases. As per participant 8, this student dropped out of the postgraduate program after this because of “ *apathetic behavior*” of professors. 

In another interview, a participant explained how professors favor some students over others in grading by either increasing or decreasing students’ grades.

**Participant 3**: *…….Those (professors) gave me a big push.*

**Interviewer** *:* *What do you mean by ‘push’?*

**Participant 3** *: We are talking about grades, giving grades*

**Interviewer** *:* *What exactly do you mean by “push”? Does he give grade with no valid reason?*

**Participant 3** *: Yeah, we are talking about the final exam, professor x has control of 40% of your grade. The grade distribution is totally unfair.*

**Interviewer** *:* *does he give grade as he wishes?*

**Participant 3** *:* *Yeah, that’s normal, that’s normal, we got used to it.*

The statement “ *that’s normal*” indicates the persistence and prevalence of the unfair grading system and students’ acceptance of that reality.

Five other participants mentioned that obedience and personal relationship with the professors could boost students’ grades.

**Interviewer:** *If a student wants the best possible grade, what is the best way to achieve that at this department?*

**Participant 5:** *Definitely not studying, definitely not studying, and definitely not clinical work. The most important thing is to stay around professors, do whatever they say. Definitely anything but studying or clinical work as we should…The best way to obtain grades is as they say…sycophancy, flattering professors or staying around them and doing what they ask you to do. And what they ask you for has nothing to do with teaching us.*

Participant repetitive use of the word “ *definitely not studying*” shows a high level of emphasis that might have stemmed from painful experience.

It is worth mentioning that Participant 5 has now dropped out of his postgraduate program and is currently pursuing his education elsewhere.

## Discussion

This study was set out to provide the first description of the clinical learning environment of the postgraduate dental education at Damascus University. To this end, in-depth interviews were conducted with 8 postgraduate students who were from every clinical department. The results suggest the serious deterioration of the CLE. First, clinical training was characterized by teachers’ inaccessibility and negligence of their teaching duties making students resort to self-reliance and informal peer-assisted learning; self-reliance was associated with dangerous learning practices such as ‘trial and error’. Second, participants reported a lack of sense of collegiality among same-year peers. Third, it was reported that the workload hindered students’ clinical training. Fourth, the adopted assessment methods were problematic on many levels; participants highlighted non-methodological, unfair and biased assessment practices, and assessment mainly played a negative and demoralizing role in which it directed students to other inappropriate approaches to earn grades rather than improving their own clinical and academic performance.

**Figure 1.**
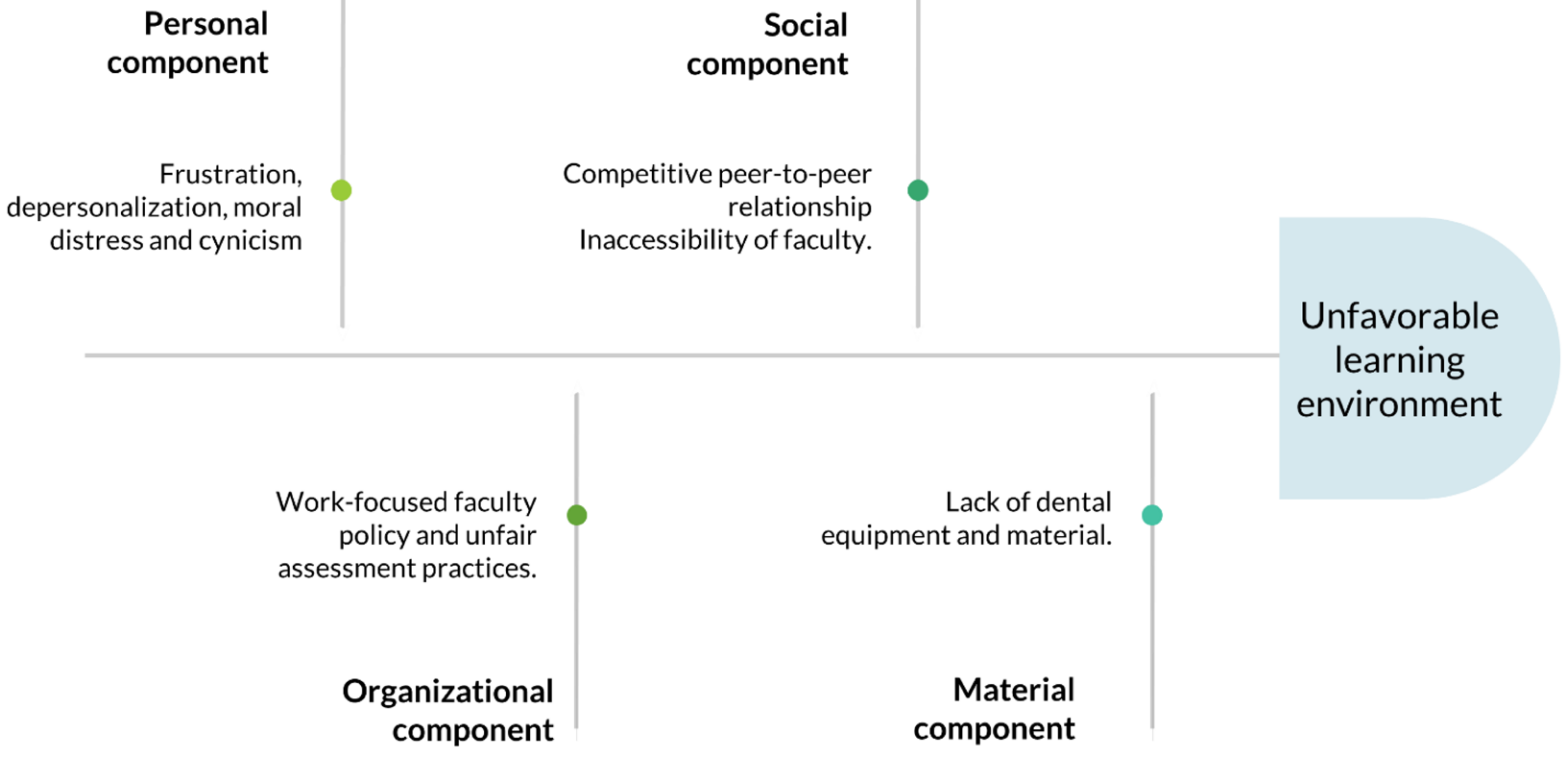
Fishbone diagram showing the four components of the learning environment and their predominant themes.

The findings of our study shed light on all components of the learning environment as conceptualized by ( Gruppen *et al*., 2019); at the psychosocial dimensions, the personal component was illustrated in how students interacted with and perceived the learning environment: students’ felt frustrated with and depersonalized by their professors; other observed adaptations students’ made with the LE is the acceptance of how professors behave and being skeptical of the intentions of their competitive peers. Positive attitude towards the state of independence experienced during clinical training also reflect the personal component. Nevertheless, individual characteristics is less likely to be the contributing problematic factor in the LE. The social component is more likely to be the major challenging aspect of the LE as the problems are widely disseminated and shared among participants. The quality of interactions between peer-to-peer appeared to be negatively perceived when it comes to same year colleagues; on the other hand, relationships between participants and their senior peers was positively perceived as they took an educational or mentor role; faculty-to-student relationship was most negative: ignoring learners’ needs, self-centeredness, poor communication and abuse of authority trust are all characteristics of the interactions of teaching staff with their students. The organizational component of the LE also appeared to be problematic as illustrated in the results where faculty policy focus on treating patients much more than teaching postgraduates; another issue in the organizational component is the used assessment system that rely on unfair criteria and methods in assessing students; another important thing to notice about the organizational component is its vagueness and variability; for example, one participant mentioned that when one professor rejects a case of his, he goes to another who would approve it. Policies and rules seem to be applied differently by different faculty members. The material dimension of the LE, the physical component in particular, was also mentioned to interfere with students’ access to learning opportunities; out of service operating rooms and lack of essential dental material and equipment disturbed the learning process by hindered students’ ability to observe and conduct certain clinical procedures. It is important to mention that our study highlights the impact of the material dimension which is understudied in the LE literature ( Gruppen *et al*., 2018). Figure 1 illustrates the aspects that led to a negatively perceived LE organized according Gruppen *et al*. conceptual framework ( Gruppen *et al*., 2019). Although the study sheds light on various components of the LE, it does that only from one angle, and this is a limitation of the current study. We have not conducted interviews with faculty members, staff nor patients. Nevertheless, students’ are key stakeholders in the educational process and therefore, their perceptions of the LE are crucial and unique; this is supported by the dental education literature ( Subramanian *et al*., 2013) which continually emphasized the importance of exploring students’ voice ( Divaris *et al*., 2008; Henzi *et al*., 2005; Schönwetter *et al*., 2006).

At an institutional level, our findings could serve as formative and constructive feedback on the supervision and teaching practices; can be included in quality assurance processes; and can empower students and encourage their democratic participation in their training journey. From a larger perspective, we believe that our findings are of value to the literature of clinical learning environment especially in compromised settings that is under-resourced and war affected. Our findings could also be transferable to other settings that have similar characteristics to Damascus University, such as using outdated curriculum and teacher-centered approaches. Hence, these findings could be used in sensitizing educators and decision makers about critical ethical issues in the CLE that must not be left unresolved. Moreover, this study sheds light on how the material dimension in LE affects students’ learning and clinical practice- an aspect that is under-represented in the LE literature ( Gruppen *et al*., 2018). Future research focusing on the interplay between the material dimension and psychosocial dimension in the LE in under-resourced settings could make use of our findings in directing and shaping their research.

Learner neglect in the clinical working environment has been considered as inevitable due to the busy nature and distractions of the work environment placed upon clinical teachers who usually have a dual role of being healthcare professionals and teachers ( Webster *et al*., 2020). Nevertheless, at Damascus University Faculty of Dental Medicine, the clinical supervisors are only responsible for teaching students; they do not have the responsibility of treating patients. The frequent unavailability of supervisors is common issue being reported previously ( Henzi *et al*., 2007; Pyle *et al*., 2006; Rudland *et al*., 2021). Faculty’s negligence of teaching duties or the lack of motivation of thereof has also been previously reported ( Divaris *et al*., 2008; Ebbeling *et al*., 2018; Rudland *et al*., 2021). The lack of teaching staff at DU in conjunction with the high number of postgraduate students could place high expectations on faculty. Faculty could be demotivated to teach due to these high expectations, the low payment and little recognition they receive from the University. In the context of learner neglect, senior peers played a positive role in mitigating this adverse experience ( Rudland *et al*., 2021); senior peers did not only provide guidance when needed but also took the role of clinical teachers when the official ones were not available. The main consequences of teacher inaccessibility in the study context were compromising patient care and the quality of students’ training; this is also supported by previous research ( Henzi *et al*., 2006; Divaris *et al*., 2008; Victoroff & Hogan, 2006). Learner neglect can have more adverse effects than abuse and humiliation which were also evident in this study ( Webster *et al*., 2020).

Similarly, other studies also described how the lack of well-equipped facilities affected dental students’ training ( Fitzgerald *et al*., 2008; Henzi *et al*., 2007). However, the lack of dental equipment at the Faculty of Dental Medicine reached a serious level in which some essential equipment, appliances, and facilities are either out of service or totally non-existent. This did not just hinder students’ learning but totally obstructed it as in the case of student pedodontists who did not receive practical training on deep sedation due to the lack of needed appliances.

Participants reported major issues in the assessment procedures implemented at the faculty. For example, the oral interview evaluation has been described as arbitrary in a sense that places great doubt in its validity and reliability. Similarly, the case presentation approach focuses on the number of cases rather than the quality of work or the variety of cases; this logbook-like method of assessing students’ clinical competence has been criticized for being unreliable ( Phillips & Jones, 2015). The findings imply a lack of transparency in the assessment criteria between students and the faculty. Biased and unfair assessment is a large component of the assessment process as suggested by participants. This way of conducting assessment could demotivate students to study, learn and hone their clinical skills and encourage other inappropriate approaches that could ensure their success in the program grade-wise such as being around professors and doing what they are told.

The findings of this qualitative study are in agreement with another quantitative study that used the DREEM (Dundee Ready Educational Environment Measure) to assess the undergraduate learning environment at Damascus University Faculty of Dental Medicine. The study revealed that undergraduate students at the clinical stage perception of the learning environment was negative and indicated that students perceived their teachers as authoritarian, the atmosphere as unpleasant and demotivating and the social self-perception as negative ( Alfakhry *et al*., 2023a); this has been found to be true in medical and pharmacy schools in Syria as well ( Alfakhry *et al*., 2022b; Alfakhry *et al*., 2023b).

It is difficult to attribute the current state of the clinical learning environment at Damascus University to one factor. It is more probable that a number of factors played a role in creating the climate which allowed such inappropriate teaching practices to fester; some of the plausible factors are: the outdated policies, the obstructive rules and regulations, lack of clear supervision and teaching protocols, lack of educationally qualified teaching staff, lack of education-focused culture, lack of education practice auditing, lack of clearly defined curriculum, lack of resources, misuse of available resources, large number of students in comparison to the faculty’s teaching capacity and the lack of motivation to make a positive change in dental education in Syria ( Alfakhry *et al*., 2020; Alfakhry *et al*., 2023a; Alfakhry *et al*., 2022a; Dashash, 2019; Kayyal & Gibbs, 2012; Kayyal & Dashash, 2010; Shehada *et al*., 2023).

There are multiple implications for the study findings. First, the reported mistreatment could cause low self-esteem, lower students’ satisfaction and increased stress and depression levels ( Richman *et al*., 1992; Schuchert, 1998; Wilkinson *et al*., 2006). Faculty negligence of their teaching duties might affect the competence of workforce. Students’ performance and skill acquisition will be limited to students’ motivation to learn, their ability to self-regulate, the availability of supportive more experienced peers and their ability to win the cooperation of their professors. The retention rate of students may decrease as shown by this study’s findings. Furthermore, the competence of these specialized dentists cannot be guaranteed and this could lead to substandard practices and limitations in their ability to deal with complex cases. This could push these students to another phase of learning by ‘trial and error’ when they run their own clinics. The ultimate consequence here is that the public dental health will suffer from the deterioration of the specialized dental workforce.

This is the first qualitative evaluation of the CLE at DU Faculty of Dental Medicine. The value of this investigations stems from the fact that this evaluation reveals hidden faulty educational practices that faculty and learners might or might not be aware of. The university strict policy does not tolerate negative feedback regarding the process of education making it even harder to register and report incidents of mistreatment of students. However, it’s the authors’ belief that raising awareness of the current areas of improvement in the CLE is one of the most important steps in addressing these issues ( Kayyal & Gibbs, 2012). It is hoped that this study will contribute to creating ‘an atmosphere of universal willingness’ among decision makers to initiate a comprehensive educational reform in dental education. The findings of this study are case specific and shed light on postgraduate student’s personal experience of the CLE at Damascus University Faculty of Dental Medicine. The reason behind using purposive sampling is that the information the study investigated is considered non-public and not all students are willing to share such information due to fear of retribution ( Chung *et al*., 2018). Therefore, we chose students who we trust to be open and honest in their answers about the reality of CLE. It is not the purpose of this study to achieve statistical generalization but rather help make analytic generalization ( Yin, 2017) which take into consideration the multilayered contextual factors that play a role in perpetuating a negative learning environment in which mistreatment, neglect and abuse of dental students are predominant.

## Conclusion

The findings of this study suggest the serious deterioration of the clinical learning environment at Damascus University Faculty of Dental Medicine. This deterioration manifested in the teaching staff’s unavailability and negligence of students, competitive social environment and unfair assessment practices. The poorly equipped facilities were another physical limitation that hinders effective clinical training. Future research should endeavor to reveal teaching staff’s perception regarding the obstacles of introducing a major reform that could improve the reality of the clinical learning environment.

## Data Availability

The interview data generated and analysed during the current study cannot be sufficiently de-identified and therefore cannot be made publicly available, due to ethical considerations. Figshare: Extended data for the research titled The Challenging Reality of the Clinical Learning Environment at Damascus University Faculty of Dental Medicine in Syria: A Qualitative Study https://doi.org/10.6084/m9.figshare.21971681.v1 ( Alfakhry & Mustafa, 2023) This project contains the following extended data: - File I Interview protocol.docx Data are available under the terms of the Creative Commons Attribution 4.0 International license (CC-BY 4.0).
