# Supplementary material for: The challenging reality of the clinical learning environment at Damascus University Faculty of Dental Medicine in Syria: A qualitative study
Source: MedEdPublish (2016). 2024 Nov 25;13:24. Originally published 2023 Apr 24. [Version 4] doi: 10.12688/mep.19564.4 (PMC11535484; doi:10.12688/mep.19564.4)
Supplement: Peer review history

# Reviewer report: Reviewer response for version 4

**DOI:** 10.21956/mep.22291.r40280

**Published:** 2024-12-23

**Authors:** Parvati Iyer

**Affiliations:** 1 University of The Pacific Arthur A. Dugoni School of Dentistry, San Francisco, California, USA

Dear authors,

 Thank you for incorporating the suggestions in your revision. The manuscript reads well and sheds light on the challenges dental schools face globally. It is suitable for indexing.

 It has more substantive points that will be valuable to the readers.

 Thank you,

 Dr. Parvati Iyer

Have any limitations of the research been acknowledged?

Yes

Is the work clearly and accurately presented and does it cite the current literature?

Yes

If applicable, is the statistical analysis and its interpretation appropriate?

Yes

Are all the source data underlying the results available to ensure full reproducibility?

Yes

Is the study design appropriate and is the work technically sound?

Yes

Are the conclusions drawn adequately supported by the results?

Yes

Are sufficient details of methods and analysis provided to allow replication by others?

Yes

Reviewer Expertise:

Educational research, educational technology

I confirm that I have read this submission and believe that I have an appropriate level of expertise to confirm that it is of an acceptable scientific standard.

# Reviewer report: Reviewer response for version 4

**DOI:** 10.21956/mep.22291.r40281

**Published:** 2024-11-28

**Authors:** Aubrie Swan Sein

**Affiliations:** 1 Columbia University, New York, New York, USA

All of the updates and comments address my critiques--the paper looks great, congratulations!

Have any limitations of the research been acknowledged?

Partly

Is the work clearly and accurately presented and does it cite the current literature?

Partly

If applicable, is the statistical analysis and its interpretation appropriate?

Not applicable

Are all the source data underlying the results available to ensure full reproducibility?

No

Is the study design appropriate and is the work technically sound?

Partly

Are the conclusions drawn adequately supported by the results?

Partly

Are sufficient details of methods and analysis provided to allow replication by others?

No

Reviewer Expertise:

Medical education; dental education; assessment for learning; academic advising

I confirm that I have read this submission and believe that I have an appropriate level of expertise to confirm that it is of an acceptable scientific standard.

# Reviewer report: Reviewer response for version 3

**DOI:** 10.21956/mep.22206.r39339

**Published:** 2024-11-15

**Authors:** Parvati Iyer

**Affiliations:** 1 University of The Pacific Arthur A. Dugoni School of Dentistry, San Francisco, California, USA

Dear authors,

 Your third version of the manuscript reads well. Please consider revising the conclusion to elucidate how your study applied the framework to tease out the setbacks of the learning environment. The current conclusion is too emotional.

 Thank you,

 Dr. Parvati Iyer

Have any limitations of the research been acknowledged?

Yes

Is the work clearly and accurately presented and does it cite the current literature?

Yes

If applicable, is the statistical analysis and its interpretation appropriate?

Yes

Are all the source data underlying the results available to ensure full reproducibility?

Yes

Is the study design appropriate and is the work technically sound?

Yes

Are the conclusions drawn adequately supported by the results?

Yes

Are sufficient details of methods and analysis provided to allow replication by others?

Yes

Reviewer Expertise:

Educational research, educational technology

I confirm that I have read this submission and believe that I have an appropriate level of expertise to confirm that it is of an acceptable scientific standard, however I have significant reservations, as outlined above.

# Response

**Published:** 2024-11-22

**Authors:** Ghaith Alfakhry

**Affiliations:** Department of Education, University of Oxford, Oxford, England, UK

**Reviewer comment:**

 Dear authors,

 Your third version of the manuscript reads well. Please consider revising the conclusion to elucidate how your study applied the framework to tease out the setbacks of the learning environment. The current conclusion is too emotional.

**Response:**

**We would like to thank Dr Iyer for the constructive and thoughtful feedback** **We have revised the conclusion in accordance to your feedback to the following (please see page 19):** **“This study utilizes Gruppen’s conceptual framework to systematically analyze the clinical learning environment in an under-resourced dental school. The findings important areas for improvement, including the unavailability of teaching staff, neglect of student needs, a competitive social atmosphere, and inconsistent assessment practices. These issues underscore the necessity for a comprehensive review of educational practices to foster a more supportive learning environment. Additionally, the inadequacy of facilities presents a critical barrier to effective clinical training, a concern relevant not only to Damascus University but also other similar under-resourced settings. Future research should focus on understanding the perspectives of teaching staff regarding the obstacles to implementing substantial reforms aimed at enhancing the clinical learning experience.”**

**The Discussion of the abstract has also been revised to the following:** **“This study employed Gruppen’s conceptual framework to conduct a theory-informed analysis of the clinical learning environment in a dental school with limited resources. The findings highlight important areas of improvement that need addressing, including the unavailability of teaching staff, neglect of student needs, a competitive social atmosphere, and inconsistent assessment practices. Future research ought to focus on understanding the perspectives of teaching faculty and decision makers regarding the obstacles to implementing substantial reforms aimed at enhancing the clinical learning experience.”**

**Reviewer comment:**

 Thank you,

 Dr. Parvati Iyer

**Response:**

**Dear reviewer, thank you for your support throughout this extensive period and your follow up insightful reviews. -The Authors**

# Reviewer report: Reviewer response for version 3

**DOI:** 10.21956/mep.22206.r39337

**Published:** 2024-11-04

**Authors:** Agnes Makhene

**Affiliations:** 1 Department of Nursing, Faculty of Health Science, University of Johannesburg, Johannesburg, South Africa

I am happy with the corrections made . The submission can be indexed.

Have any limitations of the research been acknowledged?

No

Is the work clearly and accurately presented and does it cite the current literature?

Yes

If applicable, is the statistical analysis and its interpretation appropriate?

Not applicable

Are all the source data underlying the results available to ensure full reproducibility?

Partly

Is the study design appropriate and is the work technically sound?

Yes

Are the conclusions drawn adequately supported by the results?

Yes

Are sufficient details of methods and analysis provided to allow replication by others?

Yes

Reviewer Expertise:

Qualitative research- facilitation of critical thinking both in the classroom and clinical

I confirm that I have read this submission and believe that I have an appropriate level of expertise to confirm that it is of an acceptable scientific standard.

# Reviewer report: Reviewer response for version 3

**DOI:** 10.21956/mep.22206.r39338

**Published:** 2024-10-29

**Authors:** Aubrie Swan Sein

**Affiliations:** 1 Columbia University, New York, New York, USA

I think the updates and responses to the critiques have substantially improved the paper, and have made it more like a research paper. My final critique is that I think that the conclusion paragraph overemphasizes the focus of what is happening at Damascus University… it should pertain more to the importance of reviewing and trying to improve clinical learning environments, such as that at Damascus University.  If the title was adjusted accordingly, this would help, too, otherwise this sounds like an investigative journalism report instead of a research paper. Maybe this: Challenges and Realities of the Clinical Learning Environment in Dental Education: A Case Study.

Have any limitations of the research been acknowledged?

Partly

Is the work clearly and accurately presented and does it cite the current literature?

Partly

If applicable, is the statistical analysis and its interpretation appropriate?

Not applicable

Are all the source data underlying the results available to ensure full reproducibility?

No

Is the study design appropriate and is the work technically sound?

Partly

Are the conclusions drawn adequately supported by the results?

Partly

Are sufficient details of methods and analysis provided to allow replication by others?

No

Reviewer Expertise:

Medical education; dental education; assessment for learning; academic advising

I confirm that I have read this submission and believe that I have an appropriate level of expertise to confirm that it is of an acceptable scientific standard, however I have significant reservations, as outlined above.

# Response

**Published:** 2024-11-22

**Authors:** Ghaith Alfakhry

**Affiliations:** Department of Education, University of Oxford, Oxford, England, UK

**Response:**

**We thank the Dr. Sein for the kind and constructive feedback. We have reviewed the conclusion to focus on the implications the study makes on similar under-resourced settings; please see page 19:** **“This study utilizes Gruppen’s conceptual framework to systematically analyze the clinical learning environment in an under-resourced dental school. The findings important areas for improvement, including the unavailability of teaching staff, neglect of student needs, a competitive social atmosphere, and inconsistent assessment practices. These issues underscore the necessity for a comprehensive review of educational practices to foster a more supportive learning environment. Additionally, the inadequacy of facilities presents a critical barrier to effective clinical training, a concern relevant not only to Damascus University but also other similar under-resourced settings. Future research should focus on understanding the perspectives of teaching staff regarding the obstacles to implementing substantial reforms aimed at enhancing the clinical learning experience.”** **The Discussion of the abstract has also been revised to the following:** **“This study employed Gruppen’s conceptual framework to conduct a theory-informed analysis of the clinical learning environment in a dental school with limited resources. The findings highlight important areas of improvement that need addressing, including the unavailability of teaching staff, neglect of student needs, a competitive social atmosphere, and inconsistent assessment practices. Future research ought to focus on understanding the perspectives of teaching faculty and decision makers regarding the obstacles to implementing substantial reforms aimed at enhancing the clinical learning experience.”**

**Reviewer comment:**

*If the title was adjusted accordingly, this would help, too, otherwise this sounds like an investigative journalism report instead of a research paper. Maybe this: Challenges and Realities of the Clinical Learning Environment in Dental Education: A Case Study.*

**Response:**

**We would like to extend our thanks to reviewer for the suggestion. However, the reviewer might agree with us that changing the title after about 2 years the paper has been published might not be appropriate given that it has been cited already using the current title. Furthermore, our study, like any qualitative study, is very context bound, and the findings are very particular to the setting of Damascus University and Syria. Thus, we believe it is important to mention the setting the study has been conducted. The specific target audience, in our opinion, needs to be mentioned so that interested readers in our setting can look up this paper easily. Lastly, we believe that use of “A qualitative study” distinguishes the paper from investigative journal articles.** **We would like to extend our deepest gratitude to Dr Sein for her insightful feedback which significantly helped us revise our analysis and writing. And thank you for following up with this article providing feedback as necessary. We truly appreciate it!**

**-The Authors.**

# Reviewer report: Reviewer response for version 2

**DOI:** 10.21956/mep.21148.r36022

**Published:** 2024-08-28

**Authors:** Agnes Makhene

**Affiliations:** 1 Department of Nursing, Faculty of Health Science, University of Johannesburg, Johannesburg, South Africa

The article is scientifically sound, however there is not justification of the methods chosen. The author did not described how they ensured trustworthiness of the study which is a serious omission on their part as it puts a question to the dependability, credibility, confirmability of findings as well as transferability which is a confirmation of replication of this study to other setting. Another weakness is that the authors used a lot of old sources, older than ten years and surely there is current information on the topic of this study. Lastly there is no clear description of ethical considerations in this study. I noted the authors mentioned they got written consent, however nothing is said about how they ensured confidentiality and privacy of their participants and how they managed the collected data.

Have any limitations of the research been acknowledged?

No

Is the work clearly and accurately presented and does it cite the current literature?

Yes

If applicable, is the statistical analysis and its interpretation appropriate?

Not applicable

Are all the source data underlying the results available to ensure full reproducibility?

Partly

Is the study design appropriate and is the work technically sound?

Yes

Are the conclusions drawn adequately supported by the results?

Yes

Are sufficient details of methods and analysis provided to allow replication by others?

Yes

Reviewer Expertise:

Qualitative research- facilitation of critical thinking both in the classroom and clinical

I confirm that I have read this submission and believe that I have an appropriate level of expertise to confirm that it is of an acceptable scientific standard, however I have significant reservations, as outlined above.

# Response

**Published:** 2024-10-10

**Authors:** Ghaith Alfakhry

**Affiliations:** Department of Education, University of Oxford, Oxford, England, UK

Dear Dr. Agnes,

**The authors would like to extend their honest gratitude for your insightful and accurate reflections and feedback on the study.**

***Reviewer’s Comment:***

*The article is scientifically sound, however there is no justification of the methods chosen. The author did not describe how they ensured trustworthiness of the study which is a serious omission on their part as it puts a question to the dependability, credibility, confirmability of findings as well as transferability which is a confirmation of replication of this study to other setting.*

**Response:**

**The authors would like to thank the reviewer for allowing us to elaborate on this point. Interviews were selected as the researched topic has not been studied previously so we needed to explore perceptions and understandings of the clinical learning environment. Our focus was depth rather than breadth. Our research question “How do postgraduate dental students perceive the clinical learning environment at Damascus University Faculty of Dental Medicine in Syria?” lends itself to interviews as it seeks insights, experiences, and understandings. We have added the following to the methods section, study design pg 5:** “ **Interviews were selected as the selected context has no studies exploring the clinical learning environment. The research question also lends itself to qualitative interviews as the focus is the depth of data as in exploring insights, experiences and understandings that shape participants perceptions of the clinical learning environment.**” **Regarding the trustworthiness of our findings, although we did discuss some of the issues you highlighted in the Data analysis subsection (triangulation, discussion of anomalies, consensus between authors, etc), we decided to add a subsection “Comments on rigour” to summarize some of the procedures that were done to support the trustworthiness of our findings. Please see page 7 and 8:** **“Comments on rigour** **This study followed** **Lincoln et al. (2011, p. 122)** **conceptualization of rigor which include four quality criteria: credibility, transferability, dependability, and confirmability. There are a number of practices that had been adopted to enhance trustworthiness of the findings which some were outlined briefly in the previous Methods subsections. Practices such as data triangulation and member checking support the credibility criteria. Transferability of findings was sought through thick description and discussing findings’ resonance with existing literature from different settings. Dependability was supported through achieving pragmatic saturation and iterative data generation/analysis. Lastly, good practices that we followed to ensure the confirmability of findings were: discussion of anomalies (disconfirming data/cases), discussion of research findings with other co-authors (peer-debriefing) and reflexivity regarding the researchers’ role, background and self-identifications effect on the research process and interpretation of findings.”**  

***Reviewer’s Comment:***

*Another weakness is that the authors used a lot of old sources, older than ten years and surely there is current information on the topic of this study.*

**Response:**

**Thank you for your raising this point to us. We would like to say that most of our resources are relatively recent (22 out 40) and almost all our resources are after 2000. Also, the reviewer might agree with us that what is “old” is field specific and in social sciences (including medical education) an article that is older than 10 years is not considered very old.** **Further, Damascus University Faculty of Dental Medicine curriculum is very outdated which makes the use of some old papers more relevant to our settings than new ones. For this reason, we believe that the reviewer might agree with us that the resources could be appropriate given our specific topic.**

***Reviewer’s Comment:***

*Lastly there is no clear description of ethical considerations in this study. I noted the authors mentioned they got written consent; however nothing is said about how they ensured confidentiality and privacy of their participants and how they managed the collected data.*

**Response:**

**We would like to thank the reviewer for pointing out this important aspect. We have now added more details regarding the ethical considerations we implemented to safeguard data, ensure confidentiality of participants and other good practices. Please see page 5.** **“All participants were informed of the details of the research and associated with their participation including their role, potential risks and benefits. Participants knew that their participation was voluntary and that they could withdraw from the interview at any time without giving any reason. Most interviews were conducted in private places unless the interviewer preferred otherwise. Thus, three participants were interviewed in coffee houses based on their preferences. Complete confidentiality was maintained for all participants. Any and all identifying information was redacted from the presented quotes. The translation of quotes from language into another further safeguards the identity of participants. The transcripts were securely stored in Microsoft 365 with access given to the first author only and they were not shared or published anywhere even in de-identified form to protect participants’ confidentiality from any risk even those that were very minor or unlikely. All audio files were deleted after transcribing interviews.”** **We would like to thank the reviewer again for their time, effort and expertise which they provided unconditionally.**

# Response

**Published:** 2024-10-29

**Authors:** Ghaith Alfakhry

**Affiliations:** Department of Education, University of Oxford, Oxford, England, UK

Dear Dr. Agnes,

**The authors would like to extend their honest gratitude for your insightful and accurate reflections and feedback on the study.**

**Reviewer's Comments:**

*The article is scientifically sound, however there is no justification of the methods chosen. The author did not describe how they ensured trustworthiness of the study which is a serious omission on their part as it puts a question to the dependability, credibility, confirmability of findings as well as transferability which is a confirmation of replication of this study to other setting.*

**Response:**

**The authors would like to thank the reviewer for allowing us to elaborate on this point. Interviews were selected as the researched topic has not been studied previously so we needed to explore perceptions and understandings of the clinical learning environment. Our focus was depth rather than breadth. Our research question “How do postgraduate dental students perceive the clinical learning environment at Damascus University Faculty of Dental Medicine in Syria?” lends itself to interviews as it seeks insights, experiences, and understandings. We have added the following to the methods section, study design pg 5:** “ **Interviews were selected as the selected context has no studies exploring the clinical learning environment. The research question also lends itself to qualitative interviews as the focus is the depth of data as in exploring insights, experiences and understandings that shape participants perceptions of the clinical learning environment.**” **Regarding the trustworthiness of our findings, although we did discuss some of the issues you highlighted in the Data analysis subsection (triangulation, discussion of anomalies, consensus between authors, etc), we decided to add a subsection “Comments on rigour” to summarize some of the procedures that were done to support the trustworthiness of our findings. Please see page 7 and 8:** **“Comments on rigour** **This study followed** **Lincoln et al. (2011, p. 122)** **conceptualization of rigor which include four quality criteria: credibility, transferability, dependability, and confirmability. There are a number of practices that had been adopted to enhance trustworthiness of the findings which some were outlined briefly in the previous Methods subsections. Practices such as data triangulation and member checking support the credibility criteria. Transferability of findings was sought through thick description and discussing findings’ resonance with existing literature from different settings. Dependability was supported through achieving pragmatic saturation and iterative data generation/analysis. Lastly, good practices that we followed to ensure the confirmability of findings were discussion of anomalies (disconfirming data/cases), discussion of research findings with other co-authors (peer-debriefing) and reflexivity regarding the researchers’ role, background and self-identifications effect on the research process and interpretation of findings.”**  

**Reviewer's Comments:**

*Another weakness is that the authors used a lot of old sources, older than ten years and surely there is current information on the topic of this study.*

**Response:**

**Thank you for your raising this point to us. We would like to say that most of our resources are relatively recent (22 out 40) and almost all our resources are after 2000. Also, the reviewer might agree with us that what is “old” is field specific and in social sciences (including medical education) an article that is older than 10 years is not considered very old.** **Further, Damascus University Faculty of Dental Medicine curriculum is very outdated which makes the use of some old papers more relevant to our settings than new ones. For this reason, we believe that the reviewer might agree with us that the resources could be appropriate given our specific topic.**

**Reviewer's Comments:**

*Lastly there is no clear description of ethical considerations in this study. I noted the authors mentioned they got written consent; however, nothing is said about how they ensured confidentiality and privacy of their participants and how they managed the collected data.*

**Response:**

**We would like to thank the reviewer for pointing out this important aspect. We have now added more details regarding the ethical considerations we implemented to safeguard data, ensure confidentiality of participants and other good practices. Please see page 5.** **“All participants were informed of the details of the research and associated with their participation including their role, potential risks and benefits. Participants knew that their participation was voluntary and that they could withdraw from the interview at any time without giving any reason. Most interviews were conducted in private places unless the interviewer preferred otherwise. Thus, three participants were interviewed in coffee houses based on their preferences. Complete confidentiality was maintained for all participants. Any and all identifying information was redacted from the presented quotes. The translation of quotes from language into another further safeguards the identity of participants. The transcripts were securely stored in Microsoft 365 with access given to the first author only and they were not shared or published anywhere even in de-identified form to protect participants’ confidentiality from any risk even those that were very minor or unlikely. All audio files were deleted after transcribing interviews.  ”** **We would like to thank the reviewer again for their time, effort and expertise which they provided unconditionally.**

# Reviewer report: Reviewer response for version 2

**DOI:** 10.21956/mep.21148.r36028

**Published:** 2024-04-08

**Authors:** Parvati Iyer

**Affiliations:** 1 University of The Pacific Arthur A. Dugoni School of Dentistry, San Francisco, California, USA

The revised version describes a unique challenge to dental education in difficult times and draws attention to the importance of faculty engagement with students. 

 Though the authors have incorporated several recommendations from reviewers, the manuscript could be strengthened further.

 1. The Fishbone diagram could be more detailed with sub-themes. 

 2. Future directions on how to improve the clinical learning environment from a macro and a micro factor level could be included.

 3. The conclusion could be expanded to include the sustainability of the postgraduate programs.

 Thank you,

 Reviewer

Have any limitations of the research been acknowledged?

Yes

Is the work clearly and accurately presented and does it cite the current literature?

Yes

If applicable, is the statistical analysis and its interpretation appropriate?

Yes

Are all the source data underlying the results available to ensure full reproducibility?

Yes

Is the study design appropriate and is the work technically sound?

Yes

Are the conclusions drawn adequately supported by the results?

Yes

Are sufficient details of methods and analysis provided to allow replication by others?

Yes

Reviewer Expertise:

Educational research, educational technology

I confirm that I have read this submission and believe that I have an appropriate level of expertise to confirm that it is of an acceptable scientific standard, however I have significant reservations, as outlined above.

# Response

**Published:** 2024-10-10

**Authors:** Ghaith Alfakhry

**Affiliations:** Department of Education, University of Oxford, Oxford, England, UK

**Reviewer’s Comment:** *The revised version describes a unique challenge to dental education in difficult times and draws attention to the importance of faculty engagement with students.*

*Though the authors have incorporated several recommendations from reviewers, the manuscript could be strengthened further.*  

**Response:**

**We would like to extend our gratitude and appreciation to Dr. Parvati for time and expertise she dedicated in revising our manuscript.**

1. **Reviewer’s Comment:​​​​​​​** *The Fishbone diagram could be more detailed with sub-themes. *
**Response:**
**The authors have now updated the diagram and added further details that highlight the subthemes.**
2. **Reviewer’s Comment:​​​​​​​** *Future directions on how to improve the clinical learning environment from a macro and a micro factor level could be included.*
**Response:**
** We would like to thank the reviewer for this insightful suggestion. We have now added the following paragraph to the discussion page 17 and 18:** **“Damascus University Faculty of Dental Medicine need to consider this study’s findings when planning reforms relating to their postgraduate program. Possible suggestions to improve the clinical learning environment at the macro-level is to implement specific laws and regulations that hold the faculty accountable to mis-practice, negligence of teaching duties and other expected ethical conduct. The assessment system, methods and procedures is a department that needs to major revision and safeguards to address issues of fairness and validity. The school and each department should self-regulate the assessment system in line with feasible good assessment practices. Further, we believe that students should have the right to appeal examinations where there is a possibility of lack of adherence to good assessment practices. All these macro-interventions are only possible with the actions of the school dean, vice-deans, heads of departments and other decision-makers at the dental school level. At the microlevel, we believe that every student and faculty member has the power to make a change within their circle: the faculty members could whistle blow or raise matters that need to be examined such as a certain observed mal-practice by their colleagues or by simply requesting some resources and equipment for the department. If not possible, they could do their due diligence to adhere as much as possible to good teaching practices and work on developing a caring and fair relationship with their students. Postgraduate students can also be the source of change: for example, student's groups or unions could officially flag issues in the clinical learning environment such as lack of essential equipment and the absence of clinical supervisors. They could flag these issues directly to their professors, heads of departments or other higher-level ranking faculty. It is the responsibility of the group to protect the rights of the individual. However, such practices need to be performed diligently and tactfully in a manner that does not invoke violent reactions from certain powerful political individuals. Unfortunately, within the Syrian political sphere, just and rightful objections can be misconstrued to favour the powerful and is usually responded to by unthinkable punishments reflected on those who objected.”**
3. **Reviewer’s Comment:​​​​​​​** *The conclusion could be expanded to include the sustainability of the postgraduate programs.*
**Response:**
** The authors would like to thank the reviewer for their suggestion. The conclusion has been edited to include the potential threat to the sustainability of these postgraduate programs. Please see page 18:** **Conclusion** **The findings of this study suggest the serious deterioration of the clinical learning environment at Damascus University Faculty of Dental Medicine. This deterioration manifested in the teaching staff’s unavailability and negligence of students, competitive social environment and unfair assessment practices. The poorly equipped facilities were another physical limitation that hinders effective clinical training. All of these findings raise issues that could affect the sustainability of postgraduate teaching programs. Future research should endeavour to reveal teaching staff’s perception regarding the obstacles of introducing a major reform that could improve the reality of the clinical learning environment.**
*Thank you,*
*Reviewer *

# Response

**Published:** 2024-10-29

**Authors:** Ghaith Alfakhry

**Affiliations:** Department of Education, University of Oxford, Oxford, England, UK

**Reviewer's comments:**

*The revised version describes a unique challenge to dental education in difficult times and draws attention to the importance of faculty engagement with students.*

*Though the authors have incorporated several recommendations from reviewers, the manuscript could be strengthened further.*  

**Response:**

**We would like to extend our gratitude and appreciation to Dr. Parvati for time and expertise she dedicated in revising our manuscript.**

**Reviewer's comments:**

*1. The Fishbone diagram could be more detailed with sub-themes.*

**Response:**

**The authors have now updated the diagram and added further details that highlight the subthemes.**

**Reviewer's comments:**

*2. Future directions on how to improve the clinical learning environment from a macro and a micro factor level could be included.*

**Response:**

**We would like to thank the reviewer for this insightful suggestion. We have now added the following paragraph to the discussion page 17 and 18:** **“Damascus University Faculty of Dental Medicine need to consider this study’s findings when planning reforms relating to their postgraduate program. Possible suggestions to improve the clinical learning environment at the macro-level is to implement specific laws and regulations that hold the faculty accountable to mis-practice, negligence of teaching duties and other expected ethical conduct. The assessment system, methods and procedures is a department that needs to major revision and safeguards to address issues of fairness and validity. The school and each department should self-regulate the assessment system in line with feasible good assessment practices. Further, we believe that students should have the right to appeal examinations where there is a possibility of lack of adherence to good assessment practices. All these macro-interventions are only possible with the actions of the school dean, vice-deans, heads of departments and other decision-makers at the dental school level. At the microlevel, we believe that every student and faculty member has the power to make a change within their circle: the faculty members could whistle blow or raise matters that need to be examined such as a certain observed mal-practice by their colleagues or by simply requesting some resources and equipment for the department. If not possible, they could do their due diligence to adhere as much as possible to good teaching practices and work on developing a caring and fair relationship with their students. Postgraduate students can also be the source of change: for example, students groups or unions could officially flag issues in the clinical learning environment such as lack of essential equipment and the absence of clinical supervisors. They could flag these issues directly to their professors, heads of departments or other higher-level ranking faculty. It is the responsibility of the group to protect the rights of the individual. However, such practices need to be performed diligently and tactfully in a manner that does not invoke violent reactions from certain powerful political individuals. Unfortunately, within the Syrian political sphere, just and rightful objections can be misconstrued to favour the powerful and is usually responded to by unthinkable punishments reflected on those who objected.”**

**Reviewer's comments:**

*3. The conclusion could be expanded to include the sustainability of the postgraduate programs.*

**Response:**

**The authors would like to thank the reviewer for their suggestion. The conclusion has been edited to include the potential threat to the sustainability of these postgraduate programs. Please see page 18:** **Conclusion** **The findings of this study suggest the serious deterioration of the clinical learning environment at Damascus University Faculty of Dental Medicine. This deterioration manifested in the teaching staff’s unavailability and negligence of students, competitive social environment and unfair assessment practices. The poorly equipped facilities were another physical limitation that hinders effective clinical training. All of these findings raise issues that could affect the sustainability of postgraduate teaching programs. Future research should endeavor to reveal teaching staff’s perception regarding the obstacles of introducing a major reform that could improve the reality of the clinical learning environment.**

**Reviewer's comments:**

*Thank you, Reviewer*

# Reviewer report: Reviewer response for version 2

**DOI:** 10.21956/mep.21148.r34374

**Published:** 2023-10-13

**Authors:** Aubrie Swan Sein

**Affiliations:** 1 Columbia University, New York, New York, USA

I re-read the article and there are significant improvements to it, I am impressed.

 My last suggestion is that they consider re-framing the paper as a descriptive Case Study and describe it in that way, because I think it is an appropriate description of their methods. They should also review the abstract to match the updates to the paper. I think they need a direct research question that expands on “How do postgraduate dental students perceive the clinical learning environment?” and maybe is something like: “How do postgraduate dental students perceive the clinical learning environment in an institution that has experienced 12 years of war?”

 Once these revisions are considered I can approve it.

Have any limitations of the research been acknowledged?

Partly

Is the work clearly and accurately presented and does it cite the current literature?

Partly

If applicable, is the statistical analysis and its interpretation appropriate?

Not applicable

Are all the source data underlying the results available to ensure full reproducibility?

No

Is the study design appropriate and is the work technically sound?

Partly

Are the conclusions drawn adequately supported by the results?

Partly

Are sufficient details of methods and analysis provided to allow replication by others?

No

Reviewer Expertise:

Medical education; dental education; assessment for learning; academic advising

I confirm that I have read this submission and believe that I have an appropriate level of expertise to confirm that it is of an acceptable scientific standard, however I have significant reservations, as outlined above.

# Response

**Published:** 2024-10-10

**Authors:** Ghaith Alfakhry

**Affiliations:** Department of Education, University of Oxford, Oxford, England, UK

**Reviewer’s Comment:**

*I re-read the article and there are significant improvements to it, I am impressed.*

**Response:**

**Dear Dr. Aubrie,** **the authors extend their sincere thanks for the time and effort you put in reading our revised manuscript. We also apologize for the late reply as we were waiting for other reviewers.**  

**Reviewer’s Comment:**

*My last suggestion is that they consider re-framing the paper as a descriptive Case Study and describe it in that way, because I think it is an appropriate description of their methods.*

**Response:**

**Thank you for your suggestion which we agree with. We have revised it accordingly, please see Study design, page 5:** **“This is a descriptive case study adopting an interpretivist paradigm in which reality is viewed as subjective, multiple and socially constructed.”**  

**Reviewer’s Comment:**

*They should also review the abstract to match the updates to the paper.*

**Response:**

**Thank you for your suggestion, Dr. Aubrie. As most revision was related to the way we framed our findings with Gruppen et al (2019) theory of the learning environment. We only updated the discussion section. Please see the Abstract, discussion:** **“The findings of this study were framed within Gruppen et al (2019) theory of the learning environment wherein four dimensions emerge: the personal, the social, the organizational and the material.”**

**Reviewer’s Comment:**

*I think they need a direct research question that expands on “How do postgraduate dental students perceive the clinical learning environment?” and maybe is something like: “How do postgraduate dental students perceive the clinical learning environment in an institution that has experienced 12 years of war?”*

**Response:**

**Thank you for your insight, Dr. Aubrie. Although the war definitely changed the landscape of postgraduate dental education, the interviews did not focus on war-related factors and how it affected the learning environment. We believe that the reviewer might agree with us that framing the research question as suggested might imply that our research did focus on war-related factors where in fact it did not. We did change the phrasing of the research question to highlight the context of the study which is important as the respected reviewer has suggested.** **The phrasing now is: How do postgraduate dental students perceive the clinical learning environment at Damascus University Faculty of Dental Medicine in Syria?**

**Reviewer’s Comment:**

*Once these revisions are considered I can approve it.*

**Response:**

**We hope that the revisions made rise to your expectations. If not, please let us know what we can improve. We highly appreciate the time and expertise you dedicated to helping us revise our study.**

# Response

**Published:** 2024-10-29

**Authors:** Ghaith Alfakhry

**Affiliations:** Department of Education, University of Oxford, Oxford, England, UK

**Response to reviewers:**

*I re-read the article and there are significant improvements to it, I am impressed.*

**Response:**

**Dear Dr. Aubrie,** **the authors extend their sincere thanks for the time and effort you put in reading our revised manuscript. We also apologize for the late reply as we were waiting for other reviewers.**  

**Reviewer's comments:**

*My last suggestion is that they consider re-framing the paper as a descriptive Case Study and describe it in that way, because I think it is an appropriate description of their methods.*

**Response:**

**Thank you for your suggestion which we agree with. We have revised it accordingly, please see Study design, page 5:** **“This is a descriptive case study adopting an interpretivist paradigm in which reality is viewed as subjective, multiple and socially constructed.”**  

**Reviewer's comments:**

*They should also review the abstract to match the updates to the paper.*

**Response:**

**Thank you for your suggestion, Dr. Aubrie. As most revision was related to the way we framed our findings with Gruppen et al (2019) theory of the learning environment. We only updated the discussion section. Please see the Abstract, discussion:** **“The findings of this study were framed within Gruppen et al (2019) theory of the learning environment wherein four dimensions emerge: the personal, the social, the organizational and the material.”**

**Reviewer's comments:**

*I think they need a direct research question that expands on “How do postgraduate dental students perceive the clinical learning environment?” and maybe is something like: “How do postgraduate dental students perceive the clinical learning environment in an institution that has experienced 12 years of war?”*

**Response:**

**Thank you for your insight, Dr. Aubrie. Although the war definitely changed the landscape of postgraduate dental education, the interviews did not focus on war-related factors and how it affected the learning environment. We believe that the reviewer might agree with us that framing the research question as suggested might imply that our research did focus on war-related factors where in fact it did not. We did change the phrasing of the research question to highlight the context of the study which is important as the respected reviewer has suggested.** **The phrasing now is: How do postgraduate dental students perceive the clinical learning environment at Damascus University Faculty of Dental Medicine in Syria?**

**Reviewer's comments:**

*Once these revisions are considered I can approve it.*

**Response:**

**We hope that the revisions made rise to your expectations. If not, please let us know what we can improve. We highly appreciate the time and expertise you dedicated to helping us revise our study.**

# Reviewer report: Reviewer response for version 1

**DOI:** 10.21956/mep.20958.r33524

**Published:** 2023-07-05

**Authors:** Aubrie Swan Sein

**Affiliations:** 1 Columbia University, New York, New York, USA

This project appears to be illuminating important clinical learning environment issues at one institution that are in need of systemic remedies. 

 Background: 

 This project references some of the relevant literature on describing clinical learning environments in health professions education settings, but would benefit from connection to a relevant conceptual framework. This paper by Gruppen et al 2019 presents a conceptual framework for CLE that the authors should review and consider how their work connects and builds upon this framework: Gruppen LD, Irby DM, Durning SJ, Maggio LA. Conceptualizing Learning Environments in the Health Professions. Acad Med. 2019 Jul;94(7):969-974. doi: 10.1097/ACM.0000000000002702. PMID: 30870148[ref-1]. 

 According to the Gruppen article: “First, academic journals should not publish articles on the LE without the authors having defined the LE, describing their conceptual framework and specific LE(s) under discussion. Second, researchers need to develop assessment methods that reflect the psychosocial and material dimensions to accurately assess the full dimensionality of LEs. Third, researchers need to move beyond the sole use of learner perceptions of the LE. Instead, they need to develop measures at the social and organizational level, as well as assess physical and virtual spaces. Fourth, educators need to recognize the importance of this construct and use the literature to propose and test educational innovations. Research and interventions to improve LEs suffer from the lack of conceptual clarity associated with “that which surrounds learning.” Yet, as awareness of the impact of LEs grows, there will be more efforts to understand it better, evaluate its quality, and improve it. All of these efforts will require increased clarity and rigor. Our conceptual framework is offered as a step toward that larger goal. We believe our recommendations for educators, researchers, and those involved in scholarly publication are important to optimize the LE for learners, teachers, leaders, and our patients.” 

 Method 

 The authors mention a research question in the Study Design section: “This study followed a descriptive phenomenological approach to address the research question” but I could not locate a specific research question or purpose described in the paper. What does doing a inductive qualitative investigation of the reality of the CLE at DU Faculty of Dental Medicine add to the literature clinical learning environments? It seems useful from a program evaluation perspective, and perhaps the results could be mapped onto a Qualitive Improvement fishbone diagram or something like that for internal purposes, as is, but I think the paper needs to describe more what readers and the field should take away from this project.  

 The method section shares a lot of detail, but I think that an even more thorough description of the qualitative research would be helpful, including more detail about how the qualitative approach and the research paradigm, and how codes were developed and iterated. This article might be a helpful start: O’Brien, Bridget C. PhD; Harris, Ilene B. PhD; Beckman, Thomas J. MD; Reed, Darcy A. MD, MPH; Cook, David A. MD, MHPE. Standards for Reporting Qualitative Research: A Synthesis of Recommendations. Academic Medicine 89(9):p 1245-1251, September 2014. | DOI: 10.1097/ACM.0000000000000388[ref-2] 

 Discussion 

 The authors state that “the findings of this study showcased the continuing deterioration of the clinical learning environment at Damascus University. It is hoped that these findings will encourage decision makers to introduce a comprehensive reform that addresses the curriculum, teaching practices and assessment procedures in clinical professional training.” This seems like an important finding for this dental school, but connection to a conceptual framework is needed for us to fully understand what to take away from the study.  

 Other 

 This study was approved by an IRB but I have concerns about the stakes for the participants and their colleagues given the broad publication of this study. Also, the perspectives of faculty, staff, patients and others are not included in this study, so that is a limitation.

Have any limitations of the research been acknowledged?

Partly

Is the work clearly and accurately presented and does it cite the current literature?

Partly

If applicable, is the statistical analysis and its interpretation appropriate?

Not applicable

Are all the source data underlying the results available to ensure full reproducibility?

No

Is the study design appropriate and is the work technically sound?

Partly

Are the conclusions drawn adequately supported by the results?

Partly

Are sufficient details of methods and analysis provided to allow replication by others?

No

Reviewer Expertise:

Medical education; dental education; assessment for learning; academic advising

I confirm that I have read this submission and believe that I have an appropriate level of expertise to state that I do not consider it to be of an acceptable scientific standard, for reasons outlined above.

# Response

**Published:** 2023-07-18

**Authors:** Ghaith Alfakhry

**Affiliations:** Department of Education, University of Oxford, Oxford, England, UK

Aubrie Swan

 Columbia University, New York, New York, USA

*This project appears to be illuminating important clinical learning environment issues at one*

*institution that are in need of systemic remedies.*

**Response: The authors would like to extend their thanks to the reviewer whose comments helped us improve the reporting of the manuscript substantially.**

*Background:*

*This project references some of the relevant literature on describing clinical learning*

*environments in health professions education settings, but would benefit from connection to a*

*relevant conceptual framework. This paper by Gruppen et al 2019 presents a conceptual*

*framework for CLE that the authors should review and consider how their work connects and*

*builds upon this framework: Gruppen LD, Irby DM, Durning SJ, Maggio LA. Conceptualizing*

*Learning Environments in the Health Professions. Acad Med. 2019 Jul;94(7):969-974. doi:*

*10.1097/ACM.0000000000002702. PMID: 30870148[ref-1].*

*According to the Gruppen article: “First, academic journals should not publish articles on the LE* *without the authors having defined the LE, describing their conceptual framework and specific* *LE(s) under discussion. Second, researchers need to develop assessment methods that reflect the* *psychosocial and material dimensions to accurately assess the full dimensionality of LEs. Third,* *researchers need to move beyond the sole use of learner perceptions of the LE. Instead, they need* *to develop measures at the social and organizational level, as well as assess physical and virtual* *spaces. Fourth, educators need to recognize the importance of this construct and use the* *literature to propose and test educational innovations. Research and interventions to improve Les* *suffer from the lack of conceptual clarity associated with “that which surrounds learning.” Yet, as* *awareness of the impact of LEs grows, there will be more efforts to understand it better, evaluate* *its quality, and improve it. All of these efforts will require increased clarity and rigor. Our* *conceptual framework is offered as a step toward that larger goal. We believe our* *recommendations for educators, researchers, and those involved in scholarly publication are*

*important to optimize the LE for learners, teachers, leaders, and our patients.”*

**Response: The authors would like to sincerely thank the reviewer for pointing us to a relevant conceptual framework that help us view and analyze the LE problems presented in our study. We have read Gruppen et al study in great detail and adopted their framework into our study in both the introduction, and the discussion part to illuminate and magnify the problems** **Please see Introduction, Line 52-69, page 4, 5** **:** **"** **At the Josiah Macy Jr. Foundation consensus conference, the following definition of the learning environment has been proposed: “Learning environment refers to the social interactions, organizational culture and structures, and physical and virtual spaces that surround and shape the learner’s experiences, perceptions, and learning** **(Gruppen et al., 2018)** **.” Gruppen et al has proposed a conceptual framework in which key elements of the LE has been highlighted** **(Gruppen et al., 2019)** **; the LE has been framed into five interwoven components that form two conceptual dimensions: psychosocial dimension and material dimensions; the psychosocial dimensions having three components: personal, social and organizational and the material dimension is comprised of the physical and virtual spaces. In the psychosocial component, the personal component describes how learners engage with personal growth, interact with LE, and form their own perceptions of the LE; the social component describes the relationships and social dynamics occurring within the LE such as the peer-to-peer, learner-to-faculty, and learner-to-patient relationships. The organizational component focuses on how individuals interact with organizational culture, practices, policies such as teacher control and the curriculum. The second dimension of the LE, the material dimension, encompasses the physical spaces such as buildings, hospitals, clinics and other sites that student use for learning and practice. Learning, nowadays, does not only occur within physical spaces but also in virtual ones such as electronic LEs and digital learning aids** **(Gruppen et al., 2019)** **.”**  

*Method* *The authors mention a research question in the Study Design section: “This study followed a*

*descriptive phenomenological approach to address the research question” but I could not locate a specific research question or purpose described in the paper.*

**Response: Thank you for bringing to our attention this important point. We have now explicitly stated our research question in the Method section, Line 116-118:** **“This study followed a descriptive phenomenological approach to address the research question which is phrased as follows: how do postgraduate dental students perceive the clinical learning environment?”**  

*What does doing a inductive qualitative investigation of the reality of the CLE at DU Faculty of Dental Medicine add to the literature clinical learning environments? It seems useful from a program evaluation perspective, and perhaps the results could be mapped onto a Qualitive Improvement fishbone diagram or something like that for internal purposes, as is, but I think the paper needs to describe more what readers and the field should take away from this project.*

**Response: We would like to extend our thanks to the reviewer for the insightful comments and constructive suggestions. We have now added a fishbone diagram that illustrates main themes found in the study in relation to the components of the LE as conceptualized by Gruppen et al 2019. The figure is referenced in pg 25, line 511.** ]

**Figure** **1** **.** Fishbone diagram showing the four components of the learning environment and their predominant themes.  

**As for how this study add to the literature of the clinical learning environment, we expanded on this point in pg 25-26, line 519-531:** **“At an institutional level, our findings could serve as formative and constructive feedback on the supervision and teaching practices; can be included in quality assurance processes; and can empower students and encourage their democratic participation in their training journey. From a larger perspective, we believe that our findings are of value to the literature of clinical learning environment especially in compromised settings that is under-resourced and war affected. Our findings could also be transferable to other settings that have similar characteristics to Damascus University, such as using outdated curriculum and teacher-centered approaches. Hence, these findings could be used in sensitizing educators and decision makers about critical ethical issues in the CLE that must not be left unresolved. Moreover, this study sheds light on how the material dimension in LE affects students’ learning and clinical practice- an aspect that is under-represented in the LE literature** **(Gruppen et al., 2018)** **. Future research focusing on the interplay between the material dimension and psychosocial dimension in the LE in under-resourced settings could make use of our findings in directing and shaping their research.** **”** **The claim of the transferability of our findings to other dental schools is also supported by first reviewer’s report, Arwa Alsaggaf, which stated that:**

*The issues that you are addressing in this paper are serious and dental institutes have to consider all the mentioned challenges. Being a recently graduated from different institutes in different countries (in the past two years), I saw many of that either happened to me or hear a colleague strangling with.*  

**We also briefly explained the rationale of this study from the point of view of other dental schools that share the same core characteristics as Damascus University Faculty of Dental Medicine in the Introduction, pg.6, Line 97-100:** **“The findings of this study are important for the institution itself as well as other dental schools that share core characteristics with Damascus University Faculty of Dental Medicine (war-affected, under-resourced, conventional teaching practices, teacher-centered curriculum).”** *T*

*he method section shares a lot of detail, but I think that an even more thorough description of*

*the qualitative research would be helpful, including more detail about how the qualitative*

*approach and the research paradigm, and how codes were developed and iterated. This article might be a helpful start: O’Brien, Bridget C. PhD; Harris, Ilene B. PhD; Beckman, Thomas J. MD; Reed, Darcy A. MD, MPH; Cook, David A. MD, MHPE. Standards for Reporting Qualitative Research: A Synthesis of Recommendations. Academic Medicine 89(9):p 1245-1251, September 2014. | DOI: 10.1097/ACM.0000000000000388[ref-2]*

**Response: The authors would like to sincerely thank the reviewer for the constructive feedback provided. We have read the paper mentioned above and decided to adopt it in reporting our findings.** **Research paradigm, qualitative approach are now highlighted. Please see Methods pg. 7, Line 109-118:** **“This study is reported in accordance to the Standards for Reporting Qualitative Research guidelines (O’Brien et al., 2014).** **Study design** **This study adopted an interpretivist paradigm in which reality is viewed as subjective, multiple and socially constructed. Therefore, semi-structured interviews were undertaken with postgraduate students in all clinical departments, interviews were transcribed and analyzed inductively using reflective thematic analysis. This study followed a descriptive phenomenological approach to address the research question which is phrased as follows: how do postgraduate dental students perceive the clinical learning environment?”** **The iterative process and code development was mentioned in pg. 10-11, line 198-203:** **“Interviews were deidentified during transcription process. An iterative process of collection and analysis took place. Upon the identification of potential themes, more probing questions were added to explore the themes in more depth. For example, more probing questions were added regarding the role of supervisors in teaching and their availability and accessibility during training time.** **Codes were identified inductively, as in induced from the interviews and transcripts; line by line coding strategy was implemented.** **”**

*Discussion*

*The authors state that “the findings of this study showcased the continuing deterioration of the*

*clinical learning environment at Damascus University. It is hoped that these findings will*

*encourage decision makers to introduce a comprehensive reform that addresses the curriculum, teaching practices and assessment procedures in clinical professional training.” This seems like an important finding for this dental school, but connection to a conceptual framework is needed for us to fully understand what to take away from the study.*

**Response: We would like to thank the reviewer for bringing to our attention this important point. We have now discussed our findings using the conceptual framework proposed by Gruppen et al 2019,** **Please see Discussion page 23-24, Line 485-519:** **The findings of our study shed light on all components of the learning environment as conceptualized by** **(Gruppen et al., 2019)** **; at the psychosocial dimensions, the personal component was illustrated in how students interacted with and perceived the learning environment: students’ felt frustrated with and depersonalized by their professors; other observed adaptations students’ made with the LE is the acceptance of how professors behave and being skeptical of the intentions of their competitive peers. Positive attitude towards the state of independence experienced during clinical training also reflect the personal component. Nevertheless, individual characteristics is less likely to be the contributing problematic factor in the LE. The social component is more likely to be the major challenging aspect of the LE as the problems are widely disseminated and shared among participants. The quality of interactions between peer-to-peer appeared to be negatively perceived when it comes to same year colleagues; on the other hand, relationships between participants and their senior peers was positively perceived as they took an educational or mentor role; faculty-to-student relationship was most negative: ignoring learners’ needs, self-centeredness, poor communication and abuse of authority trust are all characteristics of the interactions of teaching staff with their students. The organizational component of the LE also appeared to be problematic as illustrated in the results where faculty policy focus on treating patients much more than teaching postgraduates; another issue in the organizational component is the used assessment system that rely on unfair criteria and methods in assessing students; another important thing to notice about the organizational component is its vagueness and variability; for example, one participant mentioned that when one professor rejects a case of his, he goes to another who would approve it. Policies and rules seem to be applied differently by different faculty members. The material dimension of the LE, the physical component in particular, was also mentioned to interfere with students’ access to learning opportunities; out of service operating rooms and lack of essential dental material and equipment disturbed the learning process by hindered students’ ability to observe and conduct certain clinical procedures. It is important to mention that our study highlights the impact of the material dimension which is understudied in the LE literature** **(Gruppen et al., 2018)** **. Figure 1 illustrates the aspects that led to a negatively perceived LE organized according Gruppen et al conceptual framework** **(Gruppen et al., 2019)** **. Although the study sheds light on various components of the LE, it does that only from one angle, and this is a limitation of the current study. We have not conducted interviews with faculty members, staff nor patients.** **Nevertheless, students’ are key stakeholders in the educational process and therefore, their perceptions of the LE are crucial and unique; this is supported by the dental education literature** **(Subramanian et al., 2013)** **which continually emphasized the importance of exploring students’ voice** **(Henzi et al., 2005, Schönwetter et al., 2006, Divaris et al., 2008)** **.**

*Other*

*This study was approved by an IRB but I have concerns about the stakes for the participants and their colleagues given the broad publication of this study.*

**Response: The authors would like to thank the reviewer for the kind concern regarding this important point. As we have mentioned, this study has been approved by the review board at Damascus University Faculty of Dental Medicine** **on 17 ^th^ February 2020 (approval number: 76559).** **The participants in this study had the choice participate in this study. They were made aware that all interviews will be de-identified before analysis and no identifying information will be presented in the study. We have shared the report of the study with our participants before submitting it to MedEdPublish and they were all fine with the reporting of the interviews that took place. The research team is extremely careful about maintaining the confidentiality of our participants and we take this matter with utmost seriousness in adherence with research ethics.** **Please see pg. 10, line 183-184:** **“** **However, the final study report was shared with participants.”**** *Also, the perspectives of faculty, staff, patients and others are not included in this study, so that is a limitation.* **Response: We would like to thank the reviewer for making us aware of this limitation that we have now highlighted in the study and explained the rationale behind investigating students’ voice.** **Please see pg. 25, line 510-515:** **"** **Although the study sheds light on various components of the LE, it does that only from one angle, and this is a limitation of the current study. We have not conducted interviews with faculty members, staff nor patients. Nevertheless, students’ are key stakeholders in the educational process and therefore, their perceptions of the LE are crucial and unique; this is supported by the dental education literature** **(Subramanian et al., 2013)** **which continually emphasized the importance of exploring students’ voice** **(Henzi et al., 2005, Schönwetter et al., 2006, Divaris et al., 2008)** **."**  

**Reference**

 DIVARIS, K., BARLOW, P., CHENDEA, S., CHEONG, W., DOUNIS, A., DRAGAN, I., HAMLIN, J., HOSSEINZADEH, L., KUIN, D. & MITRIRATTANAKUL, S. 2008. The academic environment: the students’ perspective. *European Journal of Dental Education,* 12 **,** 120-130.

 GRUPPEN, L., IRBY, D. M., DURNING, S. J. & MAGGIO, L. A. 2018. Interventions designed to improve the learning environment in the health professions: a scoping review. *MedEdPublish,* 7 **,** 211.

 GRUPPEN, L. D., IRBY, D. M., DURNING, S. J. & MAGGIO, L. A. 2019. Conceptualizing learning environments in the health professions. *Academic Medicine,* 94 **,** 969-974. HENZI, D.,

 DAVIS, E., JASINEVICIUS, R., HENDRICSON, W., CINTRON, L. & ISAACS, M. 2005. Appraisal of the dental school learning environment: the students’ view. *Journal of dental education,* 69 **,** 1137-1147.

 SCHÖNWETTER, D. J., LAVIGNE, S., MAZURAT, R. & NAZARKO, O. 2006. Students’ perceptions of effective classroom and clinical teaching in dental and dental hygiene education. *Journal of Dental Education,* 70 **,** 624-635.

 SUBRAMANIAN, J., ANDERSON, V., MORGAINE, K. & THOMSON, W. 2013. The importance of ‘student voice’in dental education. *European Journal of Dental Education,* 17 **,** e136-e141.

# Reviewer report: Reviewer response for version 1

**DOI:** 10.21956/mep.20958.r33142

**Published:** 2023-05-26

**Authors:** Arwa Alsaggaf

**Affiliations:** 1 Department of Oral and Maxillofacial Surgery and Diagnostic Science, Faculty of Dentistry, Umm Al-Qura University, Mecca, Makkah Province, Saudi Arabia

Dear authors,

 The issues that you are addressing in this paper are serious and dental institutes have to consider all the mentioned challenges. Being a recently graduated from different institutes in different countries (in the past two years), I saw many of that either happened to me or hear a colleague strangling with. The CLE is an important aspect for educators and academic institutes teaching dentistry whether its undergraduate or post graduate.    . 

 Although I was excited to read it, I have a few comments/suggestions:

- The themes could've been divided differently. It could be something based on Faculty/Supervisors, Institute/Facility, Peers/Colleagues, and then sub-themes in each. It will be easier to read.
- The limitations were not clearly mentioned. 
- Finally, an expert in qualitative research might add great value to the whole paper.

Best regards,

Have any limitations of the research been acknowledged?

Partly

Is the work clearly and accurately presented and does it cite the current literature?

Yes

If applicable, is the statistical analysis and its interpretation appropriate?

I cannot comment. A qualified statistician is required.

Are all the source data underlying the results available to ensure full reproducibility?

Yes

Is the study design appropriate and is the work technically sound?

Partly

Are the conclusions drawn adequately supported by the results?

Yes

Are sufficient details of methods and analysis provided to allow replication by others?

Yes

Reviewer Expertise:

Prosthodontics, Esthetic dentistry, Clinical dentistry, in-vitro dental studies, Implant dentistry

I confirm that I have read this submission and believe that I have an appropriate level of expertise to confirm that it is of an acceptable scientific standard, however I have significant reservations, as outlined above.

# Response

**Published:** 2023-07-18

**Authors:** Ghaith Alfakhry

**Affiliations:** Department of Education, University of Oxford, Oxford, England, UK

*Arwa Alsaggaf*

*Department of Oral and Maxillofacial Surgery and Diagnostic Science, Faculty of Dentistry, Umm* *Al-Qura University, Mecca, Makkah Province, Saudi Arabia*

*Dear authors,*

*The issues that you are addressing in this paper are serious and dental institutes have to consider* *all the mentioned challenges. Being a recently graduated from different institutes in different* *countries (in the past two years), I saw many of that either happened to me or hear a colleague* *strangling with. The CLE is an important aspect for educators and academic institutes teaching* *dentistry whether its undergraduate or post graduate.* **Response:** **We would like to thank the reviewer for sharing her challenging experience with us. It is our goal to make transparent the experiences postgraduate dental students live in the clinical learning environment in the hope that this will raise the awareness of decision makers to institute new policies and rules that can optimize the CLE.**

*Although I was excited to read it, I have a few comments/suggestions:*

- *The themes could've been divided differently. It could be something based on* *Faculty/Supervisors, Institute/Facility, Peers/Colleagues, and then sub-themes in each. It will* *be easier to read.*

**Response: The authors would like to thank the reviewer for her insightful comments that we took into consideration. However, when we tried to categorize it the way suggested, some themes overlapped with others. And we think that the way each theme phrased accurately describe the collected data. Nonetheless, we have tried to re-organize the themes into these suggested themes (** ***Faculty/Supervisors, Institute/Facility, Peers/Colleagues)*** **in the discussion section.** **Please see pg. 24-25, line 493-510:** **“The social component is more likely to be the major challenging aspect of the LE as the problems are widely disseminated and shared among participants. The quality of interactions between peer-to-peer appeared to be negatively perceived when it comes to same year colleagues; on the other hand, relationships between participants and their senior peers was positively perceived as they took an educational or mentor role; faculty-to-student relationship was most negative: ignoring learners’ needs, self-centeredness, poor communication and abuse of authority trust are all characteristics of the interactions of teaching staff with their students. The organizational component of the LE also appeared to be problematic as illustrated in the results where faculty policy focus on treating patients much more than teaching postgraduates; another issue in the organizational component is the used assessment system that rely on unfair criteria and methods in assessing students; another important thing to notice about the organizational component is its vagueness and variability; for example, one participant mentioned that when one professor rejects a case of his, he goes to another who would approve it. Policies and rules seem to be applied differently by different faculty members. The material dimension of the LE, the physical component in particular, was also mentioned to interfere with students’ access to learning opportunities; out of service operating rooms and lack of essential dental material and equipment disturbed the learning process by hindered students’ ability to observe and conduct certain clinical procedures.”**  

- *The limitations were not clearly mentioned.*

**Response: We would like to thank the reviewer for pointing this out. We have now highlighted some of our limitations in the discussion section such exploring the CLE from one angle only (students’ perspective) and not investigating faculty, staff nor patient’s perspective in the study.** **Please see pg. 25, line 513-515:** **“Although the study sheds light on various components of the LE, it does that only from one angle, and this is a limitation of the current study. We have not conducted interviews with faculty members, staff nor patients.”**  

- *Finally, an expert in qualitative research might add great value to the whole paper.*

**Response: We would like to thank the reviewer for the constructive assessment of our paper. However, in Syria, besides the research team members, we did not come across any expert in qualitative research as it is an obscure field in Syria. Before starting with the project, we sought an expert in qualitative research who could help us with the design and implementation of the research; unfortunately, we did not find any. Therefore, we decided to make use of the research team knowledge qualitative research. We believe that the design, implementation and reporting was done rigorously and to the best of our abilities and that is shown in the detailed description of methods, reflexivity, coding and analysis.**

*Best regards,* **Response: The authors sincerely thank the reviewer for the insightful and comments and useful suggestions.**
